# Oxygen diffusion and vacancy migration thermally-activated govern high-temperature magnetism in ceria

**DOI:** 10.1038/s41598-019-41157-6

**Published:** 2019-03-18

**Authors:** J. Varalda, C. A. Dartora, P. C. de Camargo, A. J. A. de Oliveira, D. H. Mosca

**Affiliations:** 1 Departamento de Física, Universidade Federal do Paraná, Centro Politécnico - Caixa Postal 19044, 81531-980 Curitiba, Paraná Brazil; 2 Departamento de Engenharia Elétrica, Universidade Federal do Paraná, Centro Politécnico, 81531-980 Curitiba, Paraná Brazil; 3 Departamento de Física, Universidade Federal de São Carlos, Rod. Washington Luis, km 235 - SP-310, 13565-905 Sao Carlos, São Paulo Brazil

## Abstract

Several experimental works currently demonstrate that metallic nano-oxides and carbon nanomaterials expected to be diamagnets, in fact, behave as ferromagnets at room temperature. More than scientifically intriguing, this unconventional and unexpected ferromagnetism pave the way for innovation products and novel nanotechnological applications, gathering the magnetism to interesting functionalities of these nanomaterials. Here, we investigate the non-conventional ferromagnetism observed at high temperatures in nanocrystalline cerium dioxide (CeO_2_or nanoceria) thin films that are optically transparent to visible light. Nanoceria exhibits several concrete applications in catalytic processes, photovoltaic cells, solid-state fuel cells, among others, which are mostly due to natural presence of oxygen vacancies and easy migration of the oxygen through the structure. The ferromagnetism in non-stoichiometric nanocrystaline ceria can be consistently described by ab initio electronic structure calculations, which support that oxygen vacancies cause the formation of magnetic moments and can provide a robust interconnectivity within magnetic polarons theoretical framework. Additionally, we present a conceptual model to account the oxygen transport to the non-conventional ferromagnetism at temperatures well above room temperature. The approach is complementary to the thermally-activated effective transfers of charge and spin around oxygen vacancy centers.

## Introduction

Excellent performance of CeO_2_ - based materials for extensive applications has attracted much attention for decades^1–6^. Their physical properties come mostly from point defects consisting on missing ions (vacancies), excess ions (interstitial) or foreign kind ions (substitutional dopants). However, oxygen vacancy (V_O_) predominates among all other point defects in undoped nanoceria materials^7–9^. In particular, the wide bandgap material CeO_2_ (E_Gap_ = 3.2 to 3.6 eV) has a narrow empty band of Ce 4f states (ΔE_4f_ ~ 1 eV) and any potentially free electron created by oxygen vacancy (or doping) enters this mid-bandgap and strongly localizes onto Ce sites under small local lattice distortion. The electron trapped, by its self-induced short-range forces, in a region of the order of a lattice constant, give rise to a so-called small polaron. Indeed, such a polaron bound to a charged vacancy is also called a bound magnetic polaron, since the electrons (in this case, left behind by vacant oxygen) are localized on spin polarized Ce 4f sites^10,11^.

Point defects like normal atoms can have effective charge and spin. The meaning of effective in this case is the difference between the actual charge and spin at a lattice site and the charge and spin normally present at the site without defect. Vacancy environment (including their electrons and holes), remains almost immobile below room temperature, but can move at higher temperatures due to the thermally activated processes involving oxygen diffusion and vacancy migration.

Next, we demonstrate that room temperature ferromagnetism in nanoceria, arising from a defect chemistry dominated by V_O_ centers with polaronic-like character, is consistently described by electronic band structure calculations. Also, we settled down that the migration, of the V_O_ centers and oxygen diffusion, are both crucial to understand this unconventional ferromagnetism at high temperatures. The aim of the present work is to understand the ferromagnetism of CeO_2_ - based materials at temperatures well above room temperature, a topic that is rarely addressed in the literature.

Non-conventional ferromagnetism has been observed in many of highly insulating metallic oxides with closed-shell configuration (d° electronic configuration) that should be intrinsically diamagnets Despite some experimental and theoretical results still controversial, this non-conventional ferromagnetism is well established and universal, being found in a number of crystalline structures (including their polymorphisms) with intrinsic disorder, ranging from oxygen-deficient to highly stoichiometric oxides^12^. Non-conventional ferromagnetism is most evident in nanoscaled samples, comparatively to bulk samples. Nevertheless, a non-obvious interconnectivity between magnetic centers in defect diluted limit remains a puzzle. Although isolated point defects and their configurations often occur in the metallic oxides together with a local magnetic moment, a direct or indirect magnetic coupling mechanism between magnetic moments is not at all evident for the stabilization of the long-range ferromagnetic order^13^.

Particularly, nanoceria exhibits a defect chemistry with a strong redox activity^3^, owing to the coexistence of Ce^3+^ and Ce^4+^ oxidation states with Ce^3+^ defects and compensating V_O_. V_O_ is more abundant at the surface of a nanoparticle or at the interface of a nano-grain (or film) than in bulk^8,14^. Many of theoretical studies based on density functional theory (DFT) formalism^15^ were performed considering not only individual V_O_, but also V_O_ configurations, eventually more energetically favorable. Of course, in magnetic studies, these calculations carefully consider paramagnetic, antiferromagnetic, and ferromagnetic ground states as possible magnetic configurations. The fundamental ground state of the system is obtained by direct comparisons of global energies of these magnetic configurations.

The collection of both experimental and theoretical studies on doped and undoped nanoceria reveals some controversial results with regard to the magnitude of magnetization and a surprising interconnectivity between magnetic moments. It is also remarkable, the diversity of DFT + U methods and functional with non-single choice of U value, which primarily govern the filling of Ce 4f states^10^. Nevertheless, ferromagnetic behavior and its possible representation by *ab initio* calculations of the structure of electronic bands are clearly demonstrated^16^.

Such non-conventional ferromagnetism also reveals a robust thermal stability above room temperature. For instance, nanocrystalline thin films of undoped oxygen-defective ceria, prepared by electrodeposition technique^17,18^, consisting of a juxtaposed (111)-texture nanograins with sizes varying in the range from 5 to 10 nm, exhibit robust non-conventional ferromagnetism at room temperature^19–22^. High magnetization and peculiar saturation magnetization anisotropy results have been associated with the presence of V_O_ pairs along <111> crystallographic axes with magnetic moment greater than 2 μ_B_ per vacancy^20^.

## Results and Discussion

### Magnetic measurements

**Figure 1:**
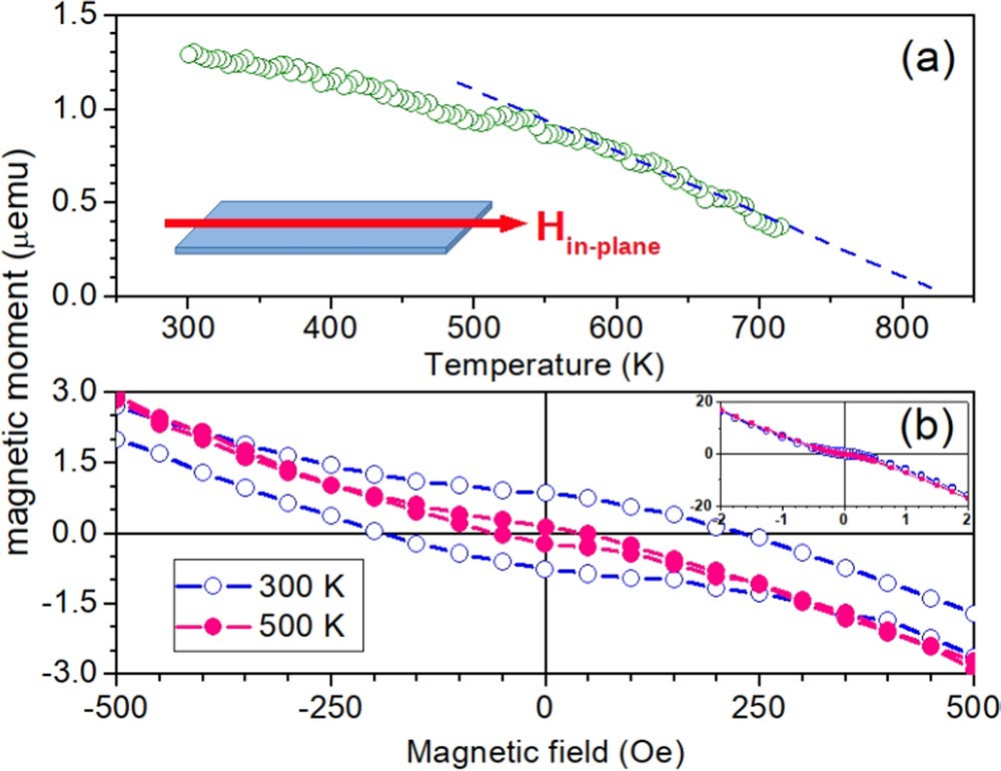
(**a**) Magnetic moment as a function of the temperature showing a fit using a law (1 − T/T_C_), which leads to an extrapolated value of T_C_ ~ 830 K. The geometry of the measurements is shown in the inset. (**b**) Magnetic hysteresis loops for a CeO_1.8_ film measured at 300 K and 500 K. In the inset in the upper right corner are shown the curves of magnetic moment versus magnetic field in units CGS given in micro-emu and kilo-oersted, respectively. Magnetic moment is shown without extraction of diamagnetic signal from substrate and sample holder.

Next, magnetic measurements of CeO_1.8_ film with characterization given in ref.^21^ are presented and discussed. Magnetic moment curves obtained during cooling down cycles at a rate of 1 K/min after diamagnetic signal extraction is shown in Fig. 1(a). A rough estimate to Curie temperature from the magnetic moment measurement can be obtained arbitrarily by assuming mean-field theory^23^. However, a good fitting of the magnetization as a function of the temperature below T_C_ is obtained by assuming an empirical (1 − T/T_C_) behavior, resulting T_C_ ~ 830 K. Surprisingly, this value is almost twice of T_C_ ~ 400 K estimated to a Ce_0.97_Co_0.03_O_1.8_ nanocrystalline film which has approximately the same saturation magnetic moment^19,24^.

Typical hysteresis loops of magnetic moment, including the diamagnetic susceptibility signal of the order −0.83 nemu/Oe owing to the sample-holder, are shown in Fig. 1(b). Clearly, an open hysteresis loop is observed for 300 K with a coercive field of 200 Oe and remnant magnetization M_R_ ~ 0.18 M_S_, where the saturation magnetization is M_S_ = 145 emu/cm^3^ (145 kA/m). Increasing temperature, the magnetization loops become progressively less hysteretic. At 500 K, coercivity is reduced to a quarter, whereas remnant magnetization is reduced by almost half. Thermal cycling procedures were repeated with different sample portions. The magnetization remains almost reversible under thermal cycles between 300 K and 700 K, in spite of a somewhat irregular noisy behavior.

Starting from the magnetic moment at room temperature, let us first consider the formation and magnitude of the magnetic moments. Roughly, CeO_1.8_ films may contain a concentration up to 20% of Ce^3+^  = [Xenon] 4f^1^, which is the source of 4f^1^ magnetic moment (*i.e*., L = 3 and S = 1/2, J = L − S = 5/2 leading to *g*J = 6/7 and *g*_J_ J = 2.14 μ_B_, where μ_B_ = 9.27 × 10^−24^ Am^2^ is the Bohr magneton.

The V_O_ migration represents a key step in the performance of CeO_2_ as an ionic conductor. In fact, CeO_2_ has attracted considerable attention for practical usage; *e.g*., catalysis and fuel cell technologies^25,26^, and even to trigger anti-apoptotic effect on a living matter under oxidative stress^27^. Below room temperature at low V_O_ density number (dilute limit), the energy barrier drastically reduces diffusion and migration processes in the bulk ceria, then surface diffusion and V_O_ hopping dominates between adjacent oxygen sites^28^. At higher concentrations, vacancies start to interfere and repel one another (dopants can also trap vacancies) at such extent that can occurs “traffic jams”, which are confirmed by a decrease in the ionic conductivity^29^. Referring to theoretical and experimental works, for undoped ceria the migration enthalpy with the lower migration barrier of the oxygen preferentially occurs along the <100> axes^30^, and along [100] and [010] directions for ceria biaxially strained. Coulomb interaction reduction owing to Ce^4+^ replacement by Ce^3+^ can alter the activation energies for V_O_ migration considerably^31^.

At higher temperatures, the first relevant snapshot is the stability of V_O_ on crystallographic planes. Theoretical and experimental results indicate that V_O_ is more stable at the surface than in the bulk and V_O_ located on a (111) surface are less stable than those on the surfaces (110) and (100)^9,32,33^. Effectively, there are more V_O_ on the (110) and (100) planes than (111), and hence a preferential alignment of V_O_ pairs occurs along <111> crystallographic axes^9,34^. Notably, the most compact oxygen-terminated surface (111) is lowest in energy, followed by (110) and (100) that come next. Then, increasing temperature the vacancy activity is distinct in these crystal planes following the order (100) > (110) > (111). Besides, because (111) planes show a higher mechanical rigidly, it implies a major blockage to the V_O_ migration^35,36^. So, starting from a random V_O_ configuration it is expected a V_O_ rearrangement with “traffic jam” along the (111) axes with increasing temperatures.

Regarding vacancy-vacancy interaction, a vacancy repels other vacancies from its nearest-neighbor shell, with the [110] and [111] directions being favorable directions for clustering of second- and third-neighbor vacancies, respectively^37^. Further, even if the excess electrons are non-randomly distributed and fully localized in a V_O_ environment, vacancies prefer not to share cations. As a consequence, V_O_ and Ce^3+^ interactions increase as the vacancy concentration increases because Ce^3+^ ions will necessarily occupy first-neighbor sites. Thus, a main direct consequence of higher V_O_ concentration is the formation of V_O_ − Ce − V_O_ bridges in the lattice^37^. Early, neutron diffraction experiments consistently indicated that Ce^4+^ ions are located in the defective V_O_ − Ce − V_O_ strings with V_O_ − V_O_ pairs ordered along the <111> axes^38^.

In regard to DFT + U calculations, there are several possible configurations of supercells starting from single V_O_ going to several V_O_ − V_O_ pairs. Typically, the magnetic moment per V_O_ is about 2 μ_B_ and magnetic interaction between V_O_ decreases with a dilution of V_O_ in the matrix^10,19^.

### Electronic band structure calculations

Several DFT + U calculations with the determination of the magnetic moment distributions (MMD) were performed for a CeO_1.5_ cell containing a V_O_-V_O_ pair along <111> crystallographic axes, which is indeed the most stable ferromagnetic configuration with the lowest total energy, and for a CeO_1.75_ cell with one isolated V_O_. These cells were obtained from the CeO_2_ basic structure (space group number 225: Fm3m) with lattice parameters *a* = 5.41 Å by removing one or two oxygen atoms along one of the <111> crystallographic axes. These two cells with specific stoichiometries and V_O_ configurations were chosen as archetypes in our analysis and discussion of magnetic percolation.

**Figure 2:**
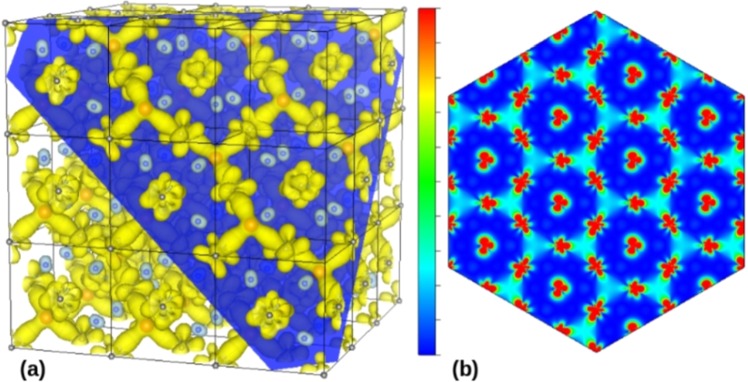
(**a**) Representative partial MMD obtained with DFT calculations for CeO_1.5_. Grey and blue spheres are respectively Ce and O atoms, whereas red spheres are representing V_O_ sites that are arranged in pairs along the <111> axis. The isosurfaces on yellow and light-blue are in opposite polarization. Only partial MMD structure consisting of the isosurface 0.0024e/Bohr^3^ is shown. For clarity, the supercell structure is interrupted by the dark-blue plane which is parallel to the (111) plane. Only the magnetic moment configuration lying on top of the (111) plane is shown. (**b**) Multi-cell global MMD map of the magnetic moment modulus in the (111) plane. The RGB (red-green-blue) colored scale varies from the minimum (blue color) to maximum (red color) values.

MMD results from the DFT calculations obtained for CeO_1.5_ are shown in Fig. 2. Figure 2(a) reveals that a percolation of the magnetic moments through V_O_ − Ce − V_O_ bridges can occur as a consequence of the hybridization of O 2p and Ce 4f orbitals, giving rise to a network with hexagonal symmetry in the (111) crystallographic plane. Such an extended electronic structure clearly favors a percolation of magnetically active Ce^3+^ ions enabling a long-range magnetic coupling to be established in the lattice. A magnetic moment is observed at O sites with inverted polarization with respect to those observed in V_O_ − Ce − V_O_ bridges. The global configuration of the magnetic moment can be visualized in a multi-cell total MMD map, as shown in Fig. 2(b). Most of the magnetic moments are found on the localized Ce 4f states, and a percolated networking of magnetic moments with weak magnitude is stabilized in semi-core states resulting in V_O_ − Ce − V_O_ bridges. These bridges have a much larger extent than the size of the Ce 4f orbital. Also, the favorable oxygen diffusion mechanism occurs along the <100> axes. A qualitatively understood comes from the fact along the oxygen rows there is considerable empty space for free oxygen diffuses. This favors the thermal activation of V_O_ migration mechanisms concomitant with a diffusion of oxygen from one point to another of the sample.

**Figure 3:**
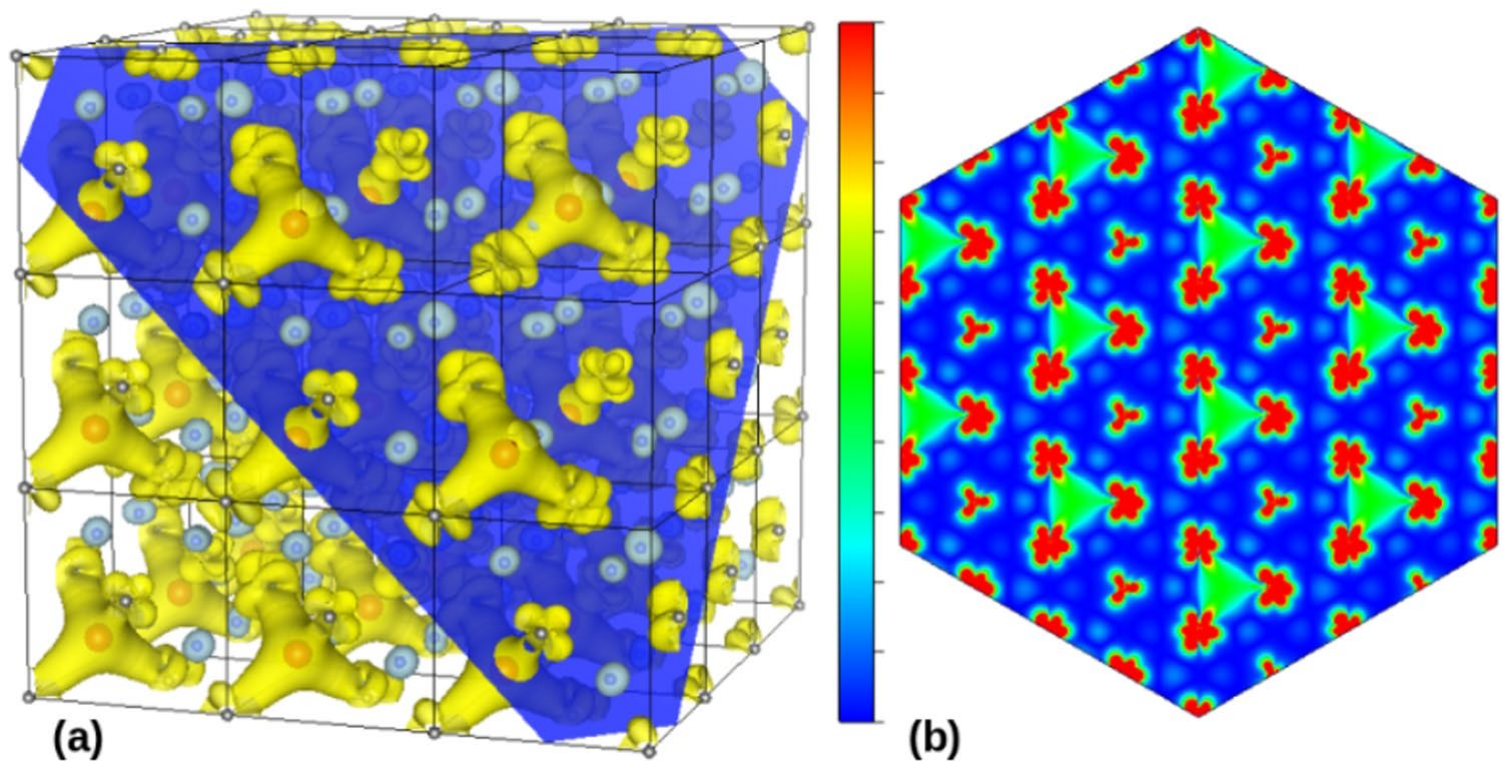
(**a**) Representative partial MMD obtained with DFT calculations for CeO_1.75_. Grey and blue spheres are respectively Ce and O atoms, whereas red spheres are representing V_O_ sites. The isosurfaces on yellow and light-blue are in opposite polarization. For clarity, the dark-blue plane cut the supercell structure in a plane parallel to (111) plane and on it can be visualized the partial MMD structure consisting of the isosurface 0.0024 e/Bohr^3^. Only the magnetic moment configuration lying on top of the (111) plane is shown. (**b**) Multi-cell global MMD map of the magnetic moment modulus in the (111) plane. Again, the RGB (red-green-blue) colored scale varies from the minimum (blue color) to maximum (red color) values.

In Fig. 3 is shown the MMD results from the DFT calculations obtained for CeO_1.75_. Representative partial MMD for CeO_1.75_ is shown in Fig. 3(a). Again, most of the magnetic moment is found on the localized Ce 4f states. However, in this case, there is no percolation through V_O_ − Ce − V_O_ bridges in the (111) planes. Spin polarized centers with a triangular shape (bound magnetic polaron) remains isolated from each other in the (111) plane, which is consistent only with a macroscopic paramagnetic behavior. This result is in clear contrast with ferromagnetic behavior observed for our sample, as shown in Fig. 1. Such an MMD is not consistent with the long-range ferromagnetic interaction persistent up to 700 K. Indeed, intrinsic electronic band structure cannot satisfactorily explain the ferromagnetism of CeO_1.75_ sample even at room temperature.

In order to emphasize this point, we remember that even arguments of V_O_ segregation that is more easily reached in the contours of the nanograins due to the preferential migration of V_O_ towards the frontiers of grains, cannot solve this problem. This assumption comes from the fact that the long-range magnetic order is strongly suppressed by thermal fluctuations in two-dimensional systems, according to Mermin-Wagner theorem. With this scenario in mind, we seek to understand the high temperature magnetization experimentally observed in highly oxygen-defective CeO_1.8_ samples consisting of (111)-textured juxtaposed nanograins. Essentially, the crucial problem is that the interconnectivity between the magnetic moment centers is ruled by magnetic polarons within crystalline structure.

### Conceptual model of magnetization at high temperatures

In order to evaluate the magnetization at high temperatures, let us consider a second snapshot that is the strength of oxygen diffusion in non-stoichiometric CeO_2_, which is well known by large scale MD simulations^39,40^. At 300 K, MD simulations indicate only significant fluctuations in the VO formation energy with quite small displacements. However, the oxygen diffusion is substantial around 1000 K, giving arises to a vacancy migration predominantly along <100> axes. The oxygen diffusion is governed by a standard Arrhenius relation:

1 $${\rm{D}}={{\rm{D}}}_{0}{\exp }(\,-\,{{\rm{E}}}_{{\rm{A}}}/\mathrm{RT})$$

where D_0_ is the temperature independent prefactor proportional to V_O_ concentration^30^, whereas E_A_ is the activation energy for vacancy jump and R = N_A_k_B_ is the universal gas constant, where N_A_ is the Avogadro constant and k_B_ = 1.38 × 10^−23^ J/K is the Boltzmann constant. The average thermal energy of an atom in the lattice is about 0.08 eV at room temperature, value usually much smaller than the activation energy (~1 eV/vacancy) and a large thermal fluctuation is needed for a jump. Experimentally, values of D_0_ = 6.2 × 10^−6^ cm^2^/sec and E_A_ = 0.16 eV/molecule-K for CeO_1.8_ are reported for temperatures below to 1000 K^40^.

Both oxygen diffusion and vacancy migration are activated by the number density gradient and temperature fluctuations. Considering that vacancies are initially concentrated at the origin as a Dirac delta function, the diffusion equation leads to a number density with a Gaussian profile as a function of position r and time t given by:

2 $${\rm{n}}({\bf{r}},{\rm{t}})={(2{\rm{\pi }}\mathrm{Dt})}^{3/2}{\exp }(\,-\,{{\rm{r}}}^{2}/2\mathrm{Dt})$$

which multiplied by r^2^ and integrated over all lattice space leads to the so-called Einstein relation for diffusion, which gives a mean value that can largely exceed one interatomic distance:

3 $$ < {{\rm{r}}}^{{\rm{2}}}\, > =6{\rm{Dt}}$$

Thus, a V_O_ can move and simulations for the migration pathway of a given oxygen when projected in the (100) planes can reach several lattice parameters in a few ns^39,40^. Specifically, oxygen can diffuse away at 800 K about 900 nm^2^ within the first millisecond. As a reference, the integration time in measurements of magnetometry and electrical conductivity is commonly set to tens of ms.

Since the vacancy migration together with oxygen diffusion process is followed by polaron formation within the diffusion area (covering a few lattice spacings in a relatively small-time scale), then magnetic percolation threshold can be reached at much lower V_O_ concentration than expected to an immobile V_O_ in CeO_2_. Using extrapolated parameters from 800 K to 300 K in Eq. (3), the migration pathway is shortened by a factor about 2.7, which can reduce to less than 10% the percolation threshold of 25% for the first nearest neighbors immobile V_O_ in CeO_2_^41^.

For CeO_2_, a sufficiently high temperature to expect significant vacancy hopping is T > Θ_D_ (Debye temperature, Θ_D_ = 480 K^42^). Additionally, the typical time interval between V_O_ jumps with subtle lattice distortions Δt must satisfies the inequality t_th_ < ζ < t_P_, where t_th_ ~ ħ/(E_A_k_B_T)^1/2^ denotes the jump-over time and t_p_ ~ ħ/W (with W the polaron energy bandwidth, which is approximately ΔE_4f_ ~ 1 eV for Ce 4f band) is the inter-site transfer time^43^. As far as, the jump-over time is much shorter than the period of a lattice vibration, an electron remains most of the time at a Ce 4f site, rarely suffering a hopping transition from site to site.

Therefore, the V_O_ motions are able to support long range magnetic coupling experimentally observed in oxygen-defective CeO_2_ samples with V_O_ number densities even below the percolation limit.

A magnetic polaron approach assisted by oxygen transport mechanism was already proposed to describe the charge transport over the oxygen-defective in CeO_2_ (111) surface^6^. However, despite a careful description of charge transport over the ceria (111) surface in the previous work, the importance of the magnetic interconnectivity was not evaluated. Additionally, it is worth noting the importance to put in evidence the formation of magnetic moment bridges for the onset of magnetic percolation (see Fig. 2), which ultimately render robust the ferromagnetism at high temperatures.

Next, we propose a model to take into account the V_O_ migration and oxygen diffusion on the magnetization dynamics at high temperatures.

In a phenomenological way, the oxygen transport and magnetization dynamics can be computed using quantum field techniques, starting from the following (thermal) Lagrangian density:

4a $$ {\mathcal L} ={ {\mathcal L} }_{0}+{ {\mathcal L} }_{{\rm{int}}}$$

4b $${ {\mathcal L} }_{0}={{\rm{\varphi }}}^{\dagger }\partial {\rm{\varphi }}/\partial \tau +({{\rm{\hslash }}}^{2}/2m)|{\boldsymbol{\nabla }}{\rm{\varphi }}{|}^{2}-({\rm{\varepsilon }}/2)|\nabla {\rm{V}}{|}^{2}-{\rm{\varepsilon }}/(2{{\rm{\lambda }}}^{2}){{\rm{V}}}^{2}$$

4c $${ {\mathcal L} }_{{\rm{int}}}=-\zeta {{\rm{\varphi }}}^{\dagger }S\,-{{\rm{\zeta }}}^{\ast }{S}^{\dagger }{\rm{\varphi }}+e{\rm{V}}{{\rm{\varphi }}}^{\dagger }{\rm{\varphi }}$$

where ϕ is a bosonic field describing free oxygen ions moving along the lattice with effective mass *m* and interacting with a screened electrostatic potential V, λ is the screening length, *S*(*S*^†^) is an annihilation (creation) field for a local magnetic moment and ζ = ħ/*τ* is an energy parameter directly related to the rate at which oxygen ions hop from one site to a neighboring vacancy, destroying the local magnetic moment, ε is the electric permittivity of the ceria, and *e* is the electric charge. In the above model, oxygen ions are relatively free to move, but they strongly interact *via* Coulomb repulsion at small distances. The interaction Lagrangian density ${ {\mathcal L} }_{{\rm{int}}}$ describes the creation of a local magnetic moment when an oxygen ion is annihilated at that site, as well as the annihilation of local magnetic moment if the site is occupied by an oxygen ion. The partition function is formulated in the language of Feynman path integral, as follows^44^:

5 $$Z=\int {\mathscr{D}}{\rm{V}}\int {\mathscr{D}}({\rm{\varphi }},{{\rm{\varphi }}}^{\dagger }){\mathscr{D}}(S,{S}^{\dagger }){\exp }(-\,\int d\tau \int dx\, {\mathcal L} )$$

where ${\mathscr{D}}{\rm{V}}$, ${\mathscr{D}}$(ϕ, ϕ^†^) and ${\mathscr{D}}$(*S*, *S*^†^)*a*re integration measures. The magnetization is obtained by the thermal average of the density operator M = μ_B_*S*^†^*S*. First, it is integrated out the electrostatic potential V, which can be done exactly by considering that the screening effect is large, *i.e*., 1/λ^2^ ≫ **q**^2^, with **q** the virtual photon momentum. Going further one can obtain the free propagator for the field ϕ, G_0_(x, y) = <(ϕ(x), ϕ^†^(y)>, which in space momentum, reads:

6 $${{\rm{G}}}_{0}({{\rm{\omega }}}_{{\rm{n}}},{\bf{k}})={[i{{\rm{\omega }}}_{{\rm{n}}},{{\rm{\hslash }}}^{2}{{\bf{k}}}^{2}/(2m)]}^{-1}$$

where ω_*n*_ = 2π*n*/β are the Matsubara energies (*n* are integer numbers) for a bosonic field at the reciprocal temperature parameter β = 1/(k_B_T). Such G_0_ is the solution of a diffusion equation, with Dirac-type source, the same which is used to obtain Eq. (2). In our simple model, the complex random dynamics of the oxygen diffusion is circumvented by taking into account only their influence in the thermodynamic average, which is suitable for magnetization measurements.

Adopting a mean field approximation for the Coulomb self-interaction of the field ϕ and integrating out this field one obtain an effective lagrangean density:

7 $${ {\mathcal L} }_{{\rm{eff}}}=|\zeta |{{\rm{S}}}^{\dagger }{[\partial /\partial \tau +({\hslash }^{{\rm{2}}}/2m){\nabla }^{2}-(2{e}^{2}{{\rm{\lambda }}}^{2}/{\rm{\varepsilon }}){{\rm{G}}}_{0}]}^{-1}{\rm{S}}$$

To obtain the average magnetization, given by M = μ_B_ <*S*^†^(β, x)*S*(0, x)>, one find in the reciprocal domain:

8 $${{\rm{M}}({\rm{\omega }}}_{{\rm{n}}},{\bf{k}})={|{\rm{\zeta }}|}^{-{\rm{2}}}{{[{\rm{G}}}_{{\rm{0}}}^{-1}-{(\mathrm{2e}}^{{\rm{2}}}{{\rm{\lambda }}}^{{\rm{2}}}{/{\rm{\varepsilon }}){\rm{G}}}_{{\rm{0}}}]}^{-{\rm{1}}}$$

and the inverse Fourier transform in d spatial dimensions of the above equation gives:

9a $${\rm{M}}({\rm{T}})={{\rm{M}}}_{0}[1-{{({\rm{T}}/{\rm{T}}}_{{\rm{C}}})}^{{\rm{d}}/2}]$$

where

9b $${{\rm{M}}}_{{\rm{0}}}={{[4(2+d){\rm{\pi }}}^{{\rm{d}}-{\rm{1}}}]}^{\mbox{--}1}{{\rm{\mu }}}_{{\rm{B}}}({{\rm{\hslash }}}^{{\rm{2}}}/m)\,{({\rm{k}}{\rm{\Theta }}}_{{\rm{D}}}/{|{\rm{\zeta }}|}^{{\rm{2}}}{){\rm{n}}}^{(1+2/{\rm{d}})}$$

and

9c $${{\rm{T}}}_{{\rm{C}}}={{\rm{K}}}_{{\rm{d}}}({\hslash }^{2}/m)[\varepsilon \,{{\rm{n}}}^{2/{\rm{d}}}\,{\hslash }^{2}/(m{{\rm{e}}}^{2}{\lambda }^{2}{)]}^{2/{\rm{d}}}/{{\rm{k}}}_{{\rm{B}}}$$

where K_d_ = 0.002 for d = 2 and K_d_ = 0.003 for d = 3. The effective density of vacancies n is introduced through the uncertainty principle, *i.e*., k_F_^n−1/d^ ~ 1, where k_F_ is the Fermi wavevector.

For d = 3, adopting n ~ 10^27^ m^−3^ for a heavy oxygen vacancy concentration, the effective mass *m* = 16 u.m.a. for oxygen atomic mass, the screening length in the range of one lattice parameter, *i.e*., λ ~ 5 Å, and permitivitty ε = 26ε_0_^16^, where ε_0_ = 8.85 × 10^−12^ F/m is the vacuum permittivity, one find a T_C_ value practically zero, indicating that the magnetic order cannot take place in the bulk.

For d = 2, which is valid to ultra-thin film geometry, we consider one CeO_2_ monolayer defined as 7.89 × 10^18^ CeO_2_ units per m^2^, which is the number of oxygen atoms per unit area in the topmost atomic layer of the CeO_2_(111) surface (taken as terminated in an open oxygen layer with a coordinated Ce^4+^ ions layer below). Dividing this by the density of bulk ceria (2.53 × 10^28^ CeO_2_ units per m^3^) gives the thickness of a CeO_2_ monolayer to be 0.31 nm. However, 20-nm-thick films have nano-grains with average sizes ranging from 5 to 10 nm^20,21^. Then, an estimate for the ratio of surface-resident atoms to atoms contained in the volume in our samples is ~1/10.

By considering n ~ 10^20^ m^−2^ and all the other values kept the same, one find T_C_ = 830 K. Notice that in d = 2 spatial dimensions the critical temperature scales with n and the magnetization falls off linearly with T following a law (1 − T/T_C_), which is in good agreement with experimental data shown in Fig. 1(a).

Therefore, the theoretical model built is capable to reproduce reasonably well the behavior of the magnetization below critical temperature considering the thermal activation of oxygen vacancies as well as oxygen migration through structure, which magnetically behaves as two-dimensional.

## Conclusions

Our present results make clear that electronic structure changes itself cannot consistently explain the non-conventional ferromagnetism in nanoceria. DFT formalism properly conducted at non-zero temperature^45^ leads to overestimated value of T_C_ ~ 2000 K. This value is just a little smaller than the melting point of 2670 K for bulk CeO_2_. However, considering the oxygen diffusional motion together with V_O_ thermal migration one can reasonably explain the long-range ferromagnetic coupling to fairly high temperatures, preserving the DFT theoretical framework, which predicts the magnetic polarization around V_O_ venters and interstitial magnetic moments bridges between Vo centres.

In summary, our theoretical approach for accounting oxygen diffusion and vacancy migration in nanocrystalline CeO_2_ thin films open a novel and deeper understanding of the high-temperature robustness of non-conventional ferromagnetism. Particularly, in nanocrystalline CeO_2_ which is an interesting nanomaterial with several proven multifunctionalities and with potential magnetic properties for innovative nanotechnological developments. Furthermore, our approach also offers a promising roadmap to exploitation of these thermal percolative mechanisms in other metal oxides.

## Methods

The detailed preparation procedure of the CeO_2_ thin films on silicon substrates, by electrodeposition technique^17^ and their nanocrystalline structure, as well as, the stoichiometry determined evaluating the amount of Ce^3+^ replacing Ce^4+^, were previously established elsewhere^18–20^. Comparisons between valence evaluations of cerium in nanoceria by X-ray photoelectron spectroscopy, electron energy loss spectroscopy and x-ray absorption near-edge spectroscopy techniques, were carefully reported^18,21^. Here, magnetic measurements correspond to the same samples reported on ref.^21^ with areas of 0.16 cm^2^ cleaved from a 20-nm-thick CeO_1.8_thin film electrodeposited on Si wafer. These films are nanocrystalline, transparent to the region of visible light, and do not generate X-ray diffractograms. According to high resolution transmission electron microscopy analyzes in the cross-sectional mode, they are formed by the juxtaposition of grains with average size varying from 5 to 10 nm, which exhibit preferential stacking of the crystalline planes (111) with small misalignments with the surface of the substrate^20^. Worth of notice is that during sample preparation it is not possible to prevent the Si surface from oxidation while CeO_2_ oxide layer coverage is formed. For this reason, a 2-nm-thick CeO_2−x_-SiO_2_ double amorphous interlayer is formed at the interface between the nanocrystalline CeO_2−x_ layer and Si substrate^21^. The presence of hydroxyl group (-OH) can not be discarded in the interlayer of CeO_2−x_-SiO_2_, because the surface of the Si wafer is hydrogen-passivated, because hydrogen atoms are fixed in its dangling bonds during HF cleaning. It is also important to recall, therefore, that in spite of CeO_2_ overlayers on the SiO_2_ substrate being quite stable (up to 1073 K), a strong loss of mass occurs around 633–723 K due to dehydroxylation of the crystal surfaces^22^. For this reason, in order to minimize the influence of the chemical changes between CeO_1.8_ film and SiO_2_ interlayer with Si substrate, magnetic measurements were performed from 300 K up to 720 K.

The DFT calculations were performed using all-electron full-potential linearized augmented-plane-wave (FP-LAPW) method, as implemented in ELK code^46^. We used U = 5 eV for Ce 4f and GGA exchange correlation functional within the PBEsol approximation^47^ for the non-collinear spin-polarized calculations. A grid of 13 × 13 × 7 k-points Brillouin zone was used for the integration in reciprocal space. The total energy and the Kohn-Sham potential convergences were better than10^−6^ Ha and 10^−8^ Ha, respectively. Some of the figures shown below were made using VESTA software^48^. The present theoretical results are *a priori* validated for single-crystals or samples with high crystalline texture at least in the scale of tens of nanometers. The stability of ferromagnetism in polycrystalline samples and in powders of different sizes is still controversial, as reported in the literature^49^.

All measurements in SQUID magnetometer start with a standard purge step procedure using a dry type primary vacuum pump. Purge steps with 99.995% ultra-pure helium gas were repeated several times and He pressures between 8 to 10Torr were used in the sample chamber during all measurements. However, this procedure does not inhibit the oxygen evolution from the sample surface in the sample chamber during the heating ramps.

In our model, oxygen vacancies are responsible for producing localized magnetic moments. The oxygen diffusion is taken into account by a charged non-relativistic bosonic scalar field to describe the ions that self-interact via electrostatic repulsion. The electrostatic potential is assumed to be screened, leading to short-range interactions, treated using a mean field. The oxygen transport dynamics is described through the Lagrangian density, given by Eqs. (4), with the partition function in a canonical ensemble, allowing us to calculate thermal properties and operator averages, written through Feynman path integrals. Therefore, the proposed Lagrangian density is plausible, and leads to usual diffusion equation in a classical limit.
